# Prevalence of Antibodies to Simbu Serogroup Viruses in Cattle in Sudan

**DOI:** 10.1155/2020/8858742

**Published:** 2020-10-20

**Authors:** Mohammed O. Hussien, Shima H. Alfaki, Khalid A. Enan, Rana A. Gafar, Amira M. Elhassan, Khalid M. Taha, Abdel Rahim M. El Hussein

**Affiliations:** ^1^Central Laboratory, Ministry of Higher Education and Scientific Research, P.O. Box 7099, Khartoum, Sudan; ^2^Central Veterinary Research Laboratory (CVRL), Animal Resources Research Corporation (ARRC), P.O. Box 8067 El Amarat, Khartoum, Sudan

## Abstract

The Simbu serogroup is one of the serogroups that belong to the *Orthobunyavirus* genus of the family *Peribunyaviridae*. Simbu serogroup viruses are transmitted mainly by *Culicoides* biting midges. Meager information is available on Simbu serogroup virus infection in ruminants in Sudan. Therefore, in this study, serological surveillance of Simbu serogroup viruses in cattle in seven states in Sudan was conducted during the period from May, 2015, to March, 2016, to shed some light on the prevalence of this group of viruses in our country. Using a cross-sectional design, 184 cattle sera were collected and tested by a commercial SBV ELISA kit which enables the detection of antibodies against various Simbu serogroup viruses. The results showed an overall 86.4% prevalence of antibodies to Simbu serogroup viruses in cattle in Sudan. Univariate analysis showed a significant association (*p*=0.007) between ELISA seropositivity and states where samples were collected. This study suggests that Simbu serogroup virus infection is present in cattle in Sudan. Further epizootiological investigations on Simbu serogroup viruses infection and virus species involved are warranted.

## 1. Introduction

The Simbu serogroup is one of the serogroups that belong to the *Orthobunyavirus* genus of the family *Peribunyaviridae*. Virus members such as Akabane virus (AKAV), Aino virus (AINOV), Sathuperi virus (SATV), Schmallenberg virus (SBV), and Shamonda virus (SHAV) are prevalent in Oceania, Australia, Africa, and Asia [1–3]. Several Simbu serogroup viruses have been shown to cross the placenta and result in outbreaks of abortion, stillbirth, and malformations [4–9]. Simbu serogroup viruses are transmitted mainly by *Culicoides* biting midges [10, 11]. The congenital malformations seen at birth are recognized as congenital arthrogryposis-hydranencephaly syndrome (CAHS) affecting the musculoskeletal and nervous systems, respectively, and related to the pregnancy stage at which the dam is infected. In cattle, severe brain deformities may happen if the dam is infected between 76 and 106 days of pregnancy [4, 10].

In Sudan, Simbu serogroup viruses such as AKAV have been reported based on serological evidence in sheep, goats, and cattle in different ecological zones [12, 13]. Elhassan et al. [13] reported a significant association (*p*=0.03) between AKAV ELISA positivity and reproductive disorders (abortion and infertility).

Owing to the meager data available on Simbu serogroup viruses infection in ruminants in Sudan, this survey was carried out to detect anti-Simbu serogroup viruses IgG antibodies in cattle sera samples obtained in seven states in Sudan during the period from May, 2015, to March, 2016.

## 2. Materials and Methods

### 2.1. Study Area

**Figure 1:**
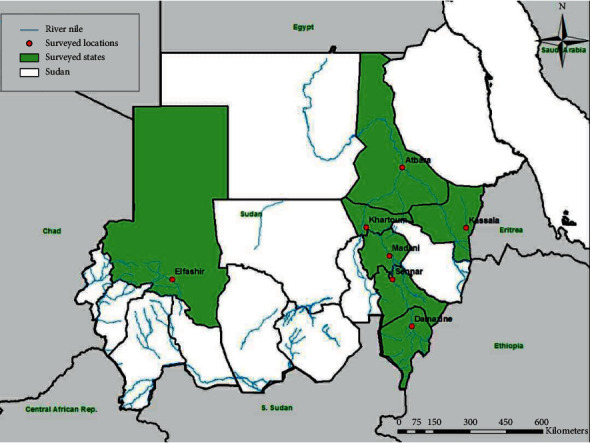
Map of Sudan showing states (green) and locations (red) where samples were collected.

The survey which was conducted in seven states in Sudan aimed to cover the most of the country. Selection of these locations was based on them being the main potential areas for livestock rearing. A cross-sectional survey that included seven states (Blue Nile (Damazine), El Gezira (Madani), Kassala (Kassala), Khartoum (Khartoum), North Darfur (Elfashir), River Nile (Atbara), and Sennar (Sennar) States) of Sudan was conducted during the period from May, 2015, to March, 2016 (Figure 1). Selection of farms was made randomly, and the formal mechanism used was lottery. In each area, samples were collected from, at least, four groups of dairy cattle that were kept apart. Collection of animal samples was reviewed and in accordance with the animal welfare code of Sudan. Five ml of blood per animal were collected from 184 adults, apparently healthy dairy cattle. Sera were obtained by centrifugation at 1500 rpm/min for 10 minutes and kept at −20°C until tested.

### 2.2. Simbu Serogroup Enzyme-linked Immunosorbent Assay (ELISA)

A commercial SBV ELISA kit (IDEXX Laboratories, USA) which enables the detection of antibodies against various Simbu serogroup viruses was used to detect anti-Simbu serogroup viruses in diluted serum samples (1/10) according to the manufacturer's instructions. The specificity and sensitivity of the ELISA kit is 99.5% and 98.1%, respectively [14]. The sample optical densities (OD) were measured by using a microplate ELISA reader (Asys Expert Plus, Austria) at 450 nm. The sample to positive control ratio (S/P ratio) was, then, determined using the formula stated in the kit brochure. The cutoff value of antibody titer is ≥40%, i.e., all samples which have an S/P ratio ≥40 are considered positive as indicated in the kit literature.

### 2.3. Statistical Analysis

Risk factor with more than two categorical levels such as state was tested individually using univariate logistic regression. Differences in antibodies to Simbu serogroup viruses between cattle and state where samples were collected were evaluated using the Chi-square (*χ*2) test. Statistical differences between all possible pairs of groups were defined as *p* < 0.05. Statistical analysis was performed using SPSS version 20 (SPSS Inc., Chicago, U.S.A.).

## 3. Results

Antibodies to Simbu serogroup viruses were detected in cattle in all areas tested with varying prevalences. The seroprevalence rates in cattle ranged from 69.2% in North Darfur to 100% in Kassala and Sennar States. The prevalence rates were the highest in Kassala and Sennar (100%), River Nile (88%), Blue Nile (85.7%), and El Gezira (83.3%) and moderate in Khartoum (76.9%) and North Darfur (69.2%) states with an overall prevalence of 86.4%. Univariable logistic regression revealed a significant association (*p*=0.007) between ELISA seropositivity and state where samples were collected (Table 1).

## 4. Discussion

There is a report of detection of antibodies to AKAV (a member of Simbu serogroup viruses) in livestock in Sudan since 1996 [12]. The present study further indicates that cattle are commonly exposed to Simbu serogroup viruses in Sudan with an overall seropositivity of 86.4%. In the current study, the overall seropositivity of Simbu serogroup viruses detected in cattle (86.4%) was lower than the estimated overall seropositivity (91.2%) of Simbu serogroup viruses reported in Nigeria [15] but was higher than that reported in cattle in Tanzania [16]. This result shows that Simbu serogroup viruses are endemic in counties in Africa that share ecological and meteorological drivers of arbovirus spread and circulation.

A high Simbu serogroup viruses seroprevalence in cattle was reported in different European countries. Seroprevalence of SBV within the herd was up to 100% and 70–100% in Germany and Netherlands, respectively [17, 18]. In the present study, the overall seropositivity of Simbu serogroup viruses is similar to the overall seropositivity of SBV in Europe: 79–94% in France [19], 90.8% in Belgium [20], and 72.5% in the Netherlands [18]. In Africa, serological screening suggests the presence of SBV in cattle, sheep, and goats in Mozambique with an overall 100% prevalence rate in cattle [21].

The differences in prevalence rates between the states herein reported may be attributed to local ecological factors, type of management and practices, flock or herd size, and insect vector activity that might influence the rates of transmission and infection with Simbu serogroup viruses. Thus, the amount of rainfall, humidity, and the plant coverage could influence the survival, abundance, and species of *Culicoides* and their activity in a specific area. Stocking rates and flock size, as well as rearing systems (grazing vs. feed lot feeding) could also affect the transmission rates of the virus (es) present in an area. Generally, arboviruses transmission and infectivity can be greatly enhanced when all of the components mentioned above in addition to immune status of host livestock and viral properties are favorable [22].

These results also support the high prevalence of AKAV (29.4%) that has previously been reported in Sudanese dairy cattle [13]. However, the much higher overall seroprevalence (86.4%) reported in the present study may indicate that the ELISA kit used is able to detect antibodies to Simbu serogroup viruses other than AKAV. This notion is supported by Oluwayelu et al. [15] who showed that all seropositive samples tested by a Simbu serogroup ELISA test were found positive for antibodies against, at least, one of the three other Simbu serogroup viruses (SBV, SHAV, and Simbu virus (SIMV)) using a serum neutralization test (SNT). Virus neutralization tests (VNT) are, thus, the best approach to distinguish antibodies against respective Simbu serogroup viruses. Otherwise, AKAV ELISA-kit results previously reported in Sudan [13] would also verify the seroprevalence of other Simbu serogroup viruses.

## 5. Conclusions

It could be concluded that Simbu serogroup viruses are widely circulating in Sudan. Finally, further epizootiological and virological investigations on Simbu serogroup viruses infection in cattle and other farm animals at the country level are important to identify the actual virus species from the vertebrate and invertebrate hosts and to determine its genetic relationships with the Simbu serogroup viruses circulating in Europe and Africa.

## Figures and Tables

**Table 1:** Univariate analysis for the association of origin of collected samples (State) and seropositivity for Simbu serogroup viruses in cattle in seven states in Sudan during the period from May, 2015, to March, 2016.

State	No of tested cattle	No positive	Prevalence rate in cattle (%)	*p* value
Blue Nile	21	18	85.7	0.007^*∗*^
El Gezira	30	25	83.3	
Kassala	30	30	100	
Khartoum	26	20	76.9	
North Darfur	26	18	69.2	
River Nile	25	22	88	
Sennar	26	26	100	
Total	184	159	86.4	

^*∗*^Significantly different

## Data Availability

Data used to support the findings of this study are available from the corresponding author upon request.
